# Ultra-minimally invasive surgery in gynecological patients: a review of the literature

**DOI:** 10.1007/s13304-022-01248-y

**Published:** 2022-04-02

**Authors:** Marco La Verde, Gaetano Riemma, Alessandro Tropea, Antonio Biondi, Stefano Cianci

**Affiliations:** 1 Department of Woman, Child and General and Specialized Surgery, Obstetrics and Gynecology Unit, University of Campania “Luigi Vanvitelli”, Largo Madonna delle Grazie 1, 80138 Naples, Italy; 2 Department for the Treatment and Study of Abdominal Diseases and Abdominal Transplantation, IRCCS-ISMETT (Istituto di Ricovero e Cura a Carattere Scientifico-Istituto Mediterraneo per i Trapianti e Terapie ad alta Specializzazione), UPMC (University of Pittsburgh Medical Center) Italy, Palermo, Italy; 3 Department of General Surgery and Medical Surgical Specialties, University of Catania, Catania, Italy; 4 Department of Obstetrics and Gynecology, University of Messina, Messina, Italy

**Keywords:** Ultra-minimally invasive, Percutaneous approach, Minimally invasive surgery, Endoscopic surgery

## Abstract

In the last decade, Ultra-minimally invasive surgery (UMIS) including both minilaparoscopic (MH) and percutaneous (PH) endoscopic surgery achieved widespread use around the world. Despite UMIS has been reported as safe and feasible surgical procedure, most of the available data are drawn from retrospective studies, with a limited number of cases and heterogeneous surgical procedures included in the analysis. This literature review aimed to analyze the most methodologically valid studies concerning major gynecological surgeries performed in UMIS. A literature review was performed double blind from January to April 2021. The keywords ‘minilaparoscopy’; ‘ultra minimally invasive surgery’; ‘3 mm’; ‘percutaneous’; and ‘Hysterectomy’ were selected in Pubmed, Medscape, Scopus, and Google scholar search engines. PRISMA (Preferred Reporting Items for Systematic reviews and Meta-Analyses) guidelines were followed for the drafting of the systematic review. The systematic literature research provided 298 studies, of which 9 fell within the inclusion criteria. Two hundred ninety-six total patients were included, 148 for both PH and MH groups. Median age (48 years), BMI (24 kg/m^2^), OT (90 min), EBL (50 ml), time to discharge (1 day), self scar evaluation (10/10), and VAS (3/10) were reported. The most frequent intraoperative complication in both the PH and MH groups was surgical bleeding. The UMIS approaches were feasible and safe even for complex gynecological procedures. Operative times and complications were superimposable to the “classical” minimally invasive approaches reported in the literature. The reported results apply only to experienced surgeons.

## Introduction

In the recent period, minimally invasive surgery (MIS) has been extensively used in all surgical specialities across the globe [1–6].

Compared to “traditional” surgical techniques, the reduced number and size of laparoscopic trocars was related to superior aesthetic results and pain tolerance while maintaining the same surgical safety [7–9].

Technological advancement has led to an increasing tendency to reduce the invasiveness of surgical experience [10–12], resulting in the establishment of a new branch of MIS, namely ultra minimally invasive surgery (UMIS), which includes both minilaparoscopic (3 mm trocar) and percutaneous endoscopic surgery [13, 14].

Suppose this trend towards a growing minimally-invasiveness is globally accepted and continuously developed in benign surgery. Minimal-invasiveness procedures also included another gynecologic area, for example, the hysteroscopic system that transitioned from a traditional approach [15, 16] to a virtual endoscopy that allows uterine cavity visualization without an invasive procedure utilizing a 3-D reconstruction [17–19].

In that case, the application of MIS in the management of gynecological malignancies must be carefully proposed in selected cases and paying attention to oncological adequacy [20–23].

The minimally invasive approach during endometrial cancer surgical staging represents the standard of care supported by the evidence of the international guidelines [24–27].

The potential of MIS during ovarian cancer surgical staging and debulking surgery [28–34] is currently under is already being investigated prospectively (Lance study) [35], whereas the discussion on its applicability to early-stage cervical cancers prompted by the LACC trial has yet to reach a consensus [34, 36–38].

Several studies [39–41] observed UMIS benefits in terms of shorter hospital stay, better aesthetic outcomes, less postoperative discomfort, and increased patient satisfaction compared to traditional laparoscopic or robotic surgery.

Furthermore, major gynecological procedures, such as percutaneous aided hysterectomy (PH) and minilaparoscopic hysterectomy (MH) using a 3 mm trocar, have been found to be safe and feasible in skilled hands [42–45].

However, most of the available data come from retrospective studies, with a small number of enrolled patients and a range of different surgical procedures included in the same research.

This literature review analyzed the most methodologically valid studies concerning major gynecological surgeries performed in UMIS. Additionally, the disadvantages and advantages of ultra-minimally intrusive techniques have been outlined.

## Materials and methods

Two authors performed a literature review double-blind from January to April 2021.

The keywords ‘minilaparoscopy’; ‘ultra minimally invasive surgery’; ‘3 mm’; ‘percutaneous’; and ‘Hysterectomy’ were selected in Pubmed, Medscape, Scopus, and Google scholar search engines.

A third author oversaw the selection of articles by the two previous authors.

All studies in English-language, with more than 15 cases reporting “complex gynecological procedures”, and performed with UMIS technique were included in the analysis.

By “complex gynecological procedures” was meant interventions included at least hysterectomy with bilateral salpingo-oophorectomy with or without pelvic lymph node dissection.

Both MH and PH have been included in the UMIS group. The minilaparoscopic surgical technique involved the placement of a 10 or 5 mm transumbilical trocar and three 3 mm ancillary trocars in the suprapubic area and the right and left flank, respectively.

The percutaneous surgical technique involved one 10 or 5 mm transumbilical optic access, one 5 mm suprapubic trocar, and two needlescopic accesses in the right and left flank.

Author, year of publication, type of device, age, body mass index (BMI), operating time (OT), estimated blood loss (EBL), day of discharge, scar patient assessment, pain visual analog scale (VAS), complication, and the type of the performed procedure were collected for each article.

Patient scar rating was determined by the patient’s subjective assessment on a scale from 0 to 10.

The VAS scale was defined as a visual pain scale ranging from 0 to 10. Complications were classified according to the Clavien-Dindo definition.

All articles not falling within the inclusion criteria, with missing data, or not related to the objective of this review were excluded.

PRISMA (Preferred Reporting Items for Systematic reviews and Meta-Analyses) [46] guidelines were followed to draft this systematic review of the literature.

## Results

The systematic literature research provided 298 studies, of which 9 fell within the inclusion criteria (3 in PH and 6 MH group) [43, 47–54].

**Fig. 1:**
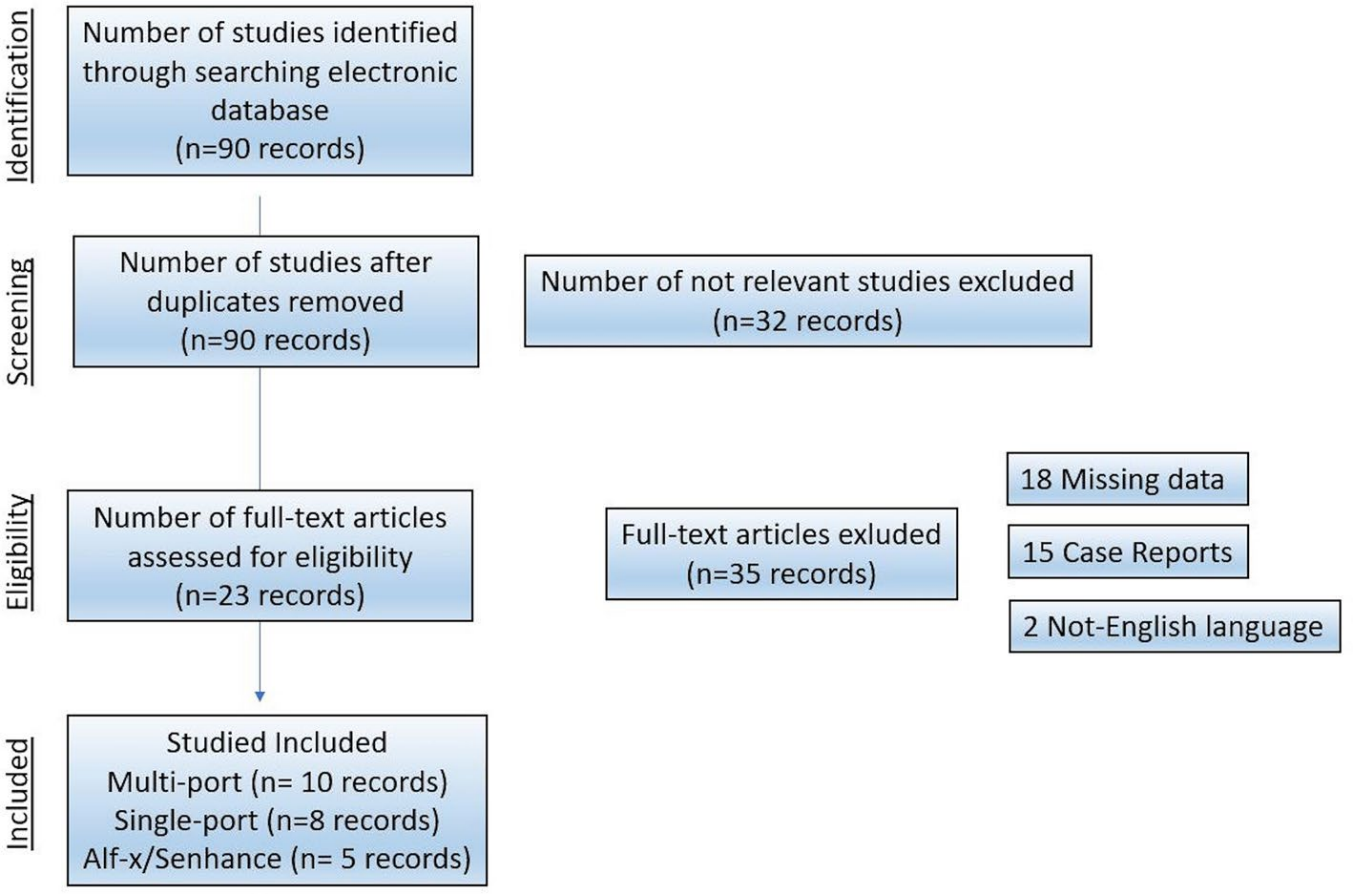
Flow diagram of the study

Ten articles were excluded because the cohort series was less than 15 patients. Eighteen case reports and 4 studies containing redundant data were excluded. One hundred and fifty-three studies did not report “complex gynecological procedures” and 111 articles did not adhere to the purpose of this review. The study selection flow chart is shown in Fig. 1. Of the included studies, 6 were retrospective in nature, one prospective, and 2 studies were randomized clinical trials.

Three studies included patients with benign disease, 4 studies involved patients with a benign disease or early-stage endometrial cancer, and 2 articles exclusively analyzed patients with malignant conditions (one included patients with early-stage endometrial cancer and the other one patients with early-stage cervical cancer).

After EC diagnosis, total hysterectomy with or without salpingo-oophorectomy were performed for all benign conditions, while nodal dissection was pursued in malignant cases [55].

Two hundred ninety-six total patients were included, 148 for both PH and MH groups.

Median age (48 years), BMI (24 kg/m^2^), OT (90 min), EBL (50 ml), time to discharge (1 day), self scar evaluation (10/10), and VAS (3/10) were reported in Tables 1 and 2 for the PH and MH group.

**Table 1:** Studies concerning single port (SP) robotic surgery

Authors, years	Type of study	Cases (number)	Surgical procedure	FIGO Stage	Operative time (min)	Ebl (ml)	Conversion rate	HS (day)	Complication (number/type)	General Outcomes	BMI (median)
Mereu et al., 2012	Retrospective study	4	Hysterectomy and salpingo-oophorectomy	2 IA 2 IB	183	50	0	2	0	SP is technically feasible and reproducible	25.7
Bogliolo et al., 2015	Prospective study	17	Hysterectomy and salpingo-oophorectomy	17 IA	171	20	0	2	4 2 Fever 1 Sciatalgic pain 1 Thromboembolism	SP is feasible and safe	32
Chung et al., 2019	Retrospective study	15	Hysterectomy, salpingo-oophorectomy, pelvic node dissection	13 IA 1 IB 1 II	155	145	0	3	1 1 Incisional hernia	SP is feasible and safe	25.4
Moukarzel et al., 2017	Retrospective cohort study	14	Hysterectomy with sentinel lymph node mapping	9 IA 1 IB 4 CAH	175	50	0	–	0	SP is cheaper than robotic multiport surgery	24.6
Moukarzel et al., 2016	Retrospective study	16	Hysterectomy with sentinel lymph node mapping	13 IA 3 CAH	175	86	1 1 Multiport: Aortic lymph node staging	1	0	SP is associated with acceptable operative times and perioperative outcomes	26
Corrado et al., 2016	Prospective study	125	Hysterectomy with or without pelvic node dissection	104 IA 19 IB 2 II	122	50	1 Not specified	2	10 2 Pelvic bleeding 2 Wound infection 2 Cystitis 1 Fever 1 Deep vein thrombosis 1 Vaginal vault hematoma 1 Lower limbs neuropathy	SP is technically feasible, safe and reproducible	27
Fagotti et al., 2013	Retrospective case–control study	19	Hysterectomy and bilateral salpingo-oophorectomy	17 IA 2 IB	90	75	0	2	1 1 Hemoperitoneum	SP is feasible and safe	26
Vizza et al., 2013	Prospective cohort trial	17	Hysterectomy and bilateral salpingo-oophorectomy	17 IA	90	75	1 1 Vaginal surgery: hypercapnia in patients with severe obesity (BMI 52)	2	0	SP is technically feasible	26.6

*CAH* complex atypical hyperplasia, *OT* operative time, *SP* single port, *HS* hospital stay, *Ebl* estimated blood loss, *BMI* body mass index

**Table 2:** Studies concerning telelap alf-x/senhance (AX/S) robotic surgery

Authors, years	Type of study	Cases number	Surgery	Stage	OT min	Ebl ml	Conversion rate	HS day	Complication number/type	Outcomes	BMI median
Gueli Alletti et al., 2018	Pilot study	10	Hysterectomy and bilateral salpingo-oophorectomy	10 IA	110	100	0	2	0	AX/S platform could be safe for hysterectomy even in obese patients	33.3
Rossitto et al., 2016	Retrospective study. Cost analysis	81	Hysterectomy, bilateral salpingo-oophorectomy with or without pelvic node dissection	81 IA	215	30	6 3 laparoscopy: hemorrhage, bladder injury, large uterine size 3 Laparotomy: large uterus, fixed uterus, anaesthesiology issue	2	2 1 bladder injury 1 severe intra-operative bleeding	AX/S robotic hysterectomy is feasible and safe and could offer specific advantages in terms of cost	–
Gueli Alletti et al., 2016	Retrospective cohort study	43	Hysterectomy, bilateral salpingo-oophorectomy with or without pelvic node dissection	43 IA	160	62	3 1 Laparoscopy: Large uterus 2 Laparotomy: severe adhesions, anaesthesiology issue	2	1 1 pelvic hematoma	AX/S approach is feasible and safe in endometrial cancer staging	25
Fanfani et al., 2015	Phase II study	44	Hysterectomy, salpingo-oophorectomy, pelvic node dissection	28 IA 16 IB	197	30	5 3 Laparoscopy: intraoperative hemorrhage, bladder injury, large uterine size 2 Laparotomy: large uterus, anesthesiology issue	2	2 1 bladder injury 1 severe intraoperative bleeding	AX/S approach is feasible and safe in endometrial cancer staging	24
Fanfani et al., 2015	Phase II study	34	Hysterectomy, salpingo-oophorectomy, pelvic node dissection	34 IA	160	50	3 1 Laparoscopy: intraoperative bleeding 2 Laparotomy: Large uterine size, anesthesiology issue	2	0	AX/S is feasible and safe	23.7

*OT* operative time, *HS* hospital stay, *Ebl* estimated blood loss, *AX/S* telelap alf-x/senhance, *BMI* body mass index

As shown in Table 3, 21 total complications were reported, 2 intraoperative and 6 postoperative in the PH group, and 5 intraoperative and 8 postoperative in the MH group.

**Table 3:** Studies concerning Multi-port (MP) Robotic surgery

Authors, years	Type of study	Cases number	Surgery	Stage	OT min	Ebl ml	Conversion rate	HS day	Complication number/type	Outcomes	BMI median
Corrado et al., 2018	Retrospective multi-institutional study	249	Hysterectomy, salpingo-oophorectomy, pelvic node dissection	153 IA 58 IB 18 II 8 IIIA 2 IIIB 8 IIIC 2 IVB	183	124	8 6 Laparoscopy: 3 hypercapnia, poor exposure, large uterus, difficulty to perform lymphadenectomy 2 Laparotomy: poor bowel exposure, bowel adhesion	3.1	24 1 Hemoperitoneum, 1 urethrovaginal fistula Others cases not specified	MP robotic surgery in severely obese women with endometrial cancer is feasible, safe, and reproducible	36.3
Yim et al., 2015	Retrospective study	112	Hysterectomy, salpingo-oophorectomy, pelvic node dissection	97 I 7 II 8 III Not specified	208	184	0	8.9	8 3 Vessel injury, 1 Febrile morbidity, 2 Pelvic cavity infection/hematoma, 1 Massive chyle ascites, 1 Wound infection	MP robot-assisted laparoscopic surgery is a feasible approach in gynecology with acceptable complications	23
Al-Badawi et al., 2011	Retrospective study	12	Hysterectomy, bilateral salpingo-oophorectomy with or without pelvic node dissection	Not specified	156	177	1 1 Laparotomy: bleeding	3.3	2 1 Post-operative bleeding, 1 supra-ventricular tachycardia	MP robotic surgery is feasible and satisfactory to our Arabian patient population	34
Smith et al., 2012	Retrospective study	46	Hysterectomy, bilateral salpingo-oophorectomy with or without pelvic node dissection	Not specified	175	94	3 3 Laparotomy: 2 intact specimen extraction, bleeding	1.3	2 1 Vascular injury, 1 deep vein thrombosis	Incorporating fellow education into MP robotic surgery does not adversely affect outcomes when compared to traditional laparoscopic surgery	30
Holloway et al., 2012	Retrospective study	35	Hysterectomy, salpingo-oophorectomy, pelvic node dissection	9 Low-risk 26 High-risk Not specified	169	118	0	1.3	0	Fluorescence imaging with indocyanine green detected bilateral sentinel lymph nodes more often than isosulfan blue	33.1
Ng et al., 2011	Retrospective study	17	Hysterectomy, salpingo-oophorectomy, with or without pelvic node dissection	Not specified	200	–	0	–	2 1 Vaginal cuff dehiscence, 1 bleeding	MP robotic surgery is feasible and safe	–
Goel et al., 2011	Retrospective study	59	Hysterectomy, salpingo-oophorectomy, with or without pelvic and aortic node dissection	18 IA 21 IB 12 II 2 III A 8 III C	185	231	1 1 Laparotomy: injury to the external iliac vein	1.3	2 1 Injury to the external iliac vein, 1 pelvic abscess	MP robotic surgery is a useful minimally invasive tool for the comprehensive surgical staging	39.3
Peeters et al., 2011	Prospective study	171	Hysterectomy, salpingo-oophorectomy, pelvic node dissection, with or without aortic node dissection	122 I 16 II 24 III 3 IV 6 CAH	49 (only operative time reported)	87	6 6 Minilaparotomy: to remove the uterus	1.4	4 4 wound complications	Minor technical and surgical approaches were associated with low morbidity, and appears to benefit patients undergoing MP robotic surgery for gynaecologic cancers	31.6
Holloway et al., 2009	Retrospective chart review	100	Hysterectomy, salpingo-oophorectomy, pelvic node dissection, with or without aortic node dissection	79 I 7 II 14 III Not specified	171	103	4 4 Laparotomy: 2 vena cava bleeding, large uterus, external iliac artery bleeding	1.1	3 1 fever, 1 postoperative ileus, 1 respiratory failure	Operative times decreased and aortic dissections improved with increasing Lymph nodes counts during the first 100 cases of MP robotic hysterectomy	29
Peiretti et al., 2009	Prospective study	80	Hysterectomy, salpingo-oophorectomy, with or without pelvic and aortic node dissection	62 IA 9 IB 2 II 3 IIIA 1 IIIB 3 IIIC	181	44	3 3 Laparotomy: 2 extensive adhesions, metastatic obturator node	2.5	5 1 Bladder fistula, 3 vaginal cuff dehiscence, 1 small bowel obstruction	MP robotic staging for early-stage endometrial cancer is feasible and safe	25.2

*OT* operative time, *HS* hospital stay, *Ebl* estimated blood loss, *MP* multi port, *BMI* body mass index

The most frequent intraoperative complication in both the PH and MH groups was surgical bleeding (6 cases out of 7 total intraoperative complications). The most commonly reported postoperative complications were bleeding (3 cases), fever (3 cases), and urinary infection (2 cases). All complications were managed with conservative treatment and were classified as Dindo grade 1 or 2.

## Discussion and evidence synthesis

Based on the main findings of the literature we stratified the discussion by focusing on the strengths and weaknesses of the UMIS technique.

## Strengths

### Cosmetic outcomes

Since its introduction in 1998, UMIS was aimed to reduce the size of abdominal scars while simultaneously increasing the quality of life of patients [56].

According to subjective patient perception [57], there is no doubt that the decreased width of the surgical scar in both the PH and the MH groups resulted in superior aesthetic outcomes.

The percutaneous method, in particular, is regarded as the greatest example of “scarless surgery,” with the surgical scar reported on postoperative day 30 as scarcely discernible [58].

In our analysis, all patients showed an extremely high level of cosmetic satisfaction.

Similar results were also obtained for other general and urologic surgeries [59, 60]. Furthermore, as reported by David et al. [61], the same excellent cosmetic outcomes could be achieved for complex upper abdominal procedures.

The effects of abdominal surgical scars had received less attention than those of face surgical scars [36, 54], even though they might have significant physical and psychological consequences [44, 62].

Furthermore, further clinical studies are required to evaluate and further analyze the psychological influence of the abdominal scar on patients’ quality of life [63, 64] in this context.

### Pain relief

Excellent pain management was noted in the patients included in the analysis, with a median “mild pain” reflected at the VAS score (VAS score 1–3 defines “mild pain”). These findings are supported by a large amount of scientific research, which includes both the UMIS and the MIS approaches [65–68].

Donnez et al. [69] found a mean VAS score of 4 (3.5 2.6) at 1 h following surgery in MIS hysterectomy patients.

**Fig. 2:**
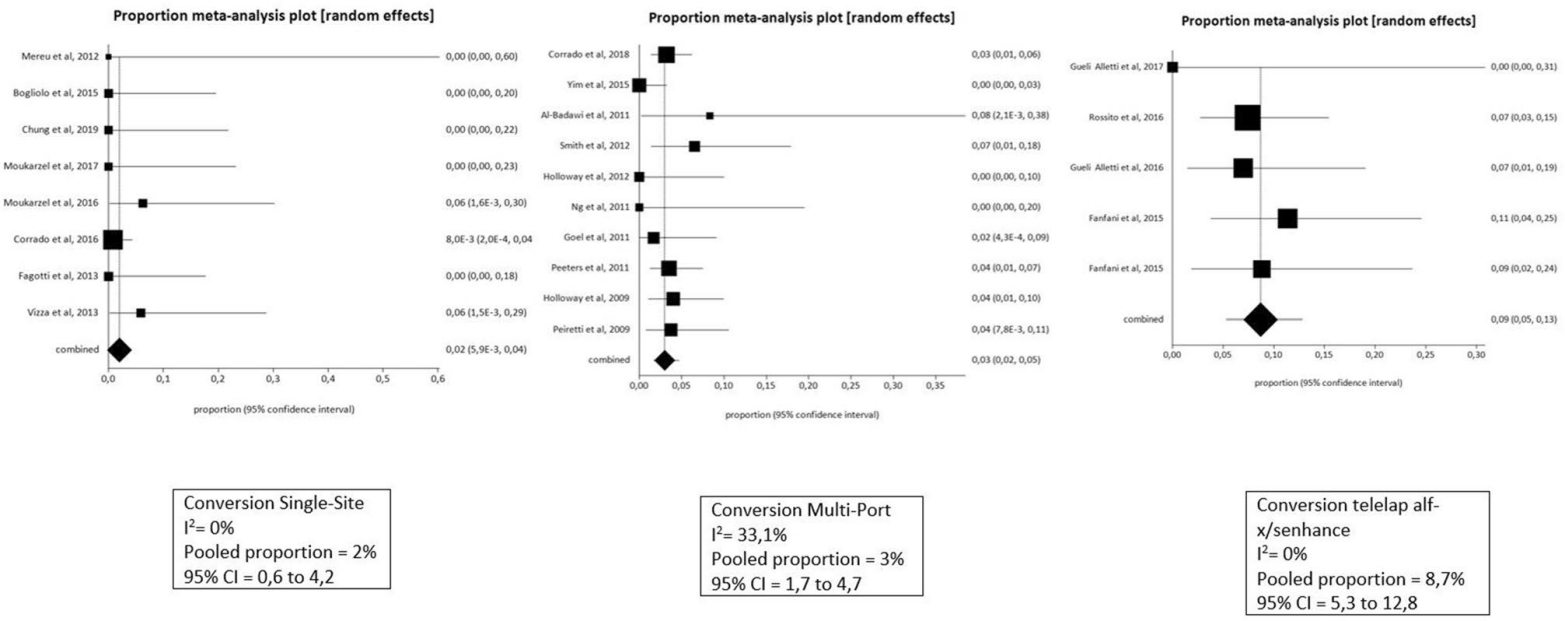
Pooled analysis for laparotomic conversions

**Fig. 3:**
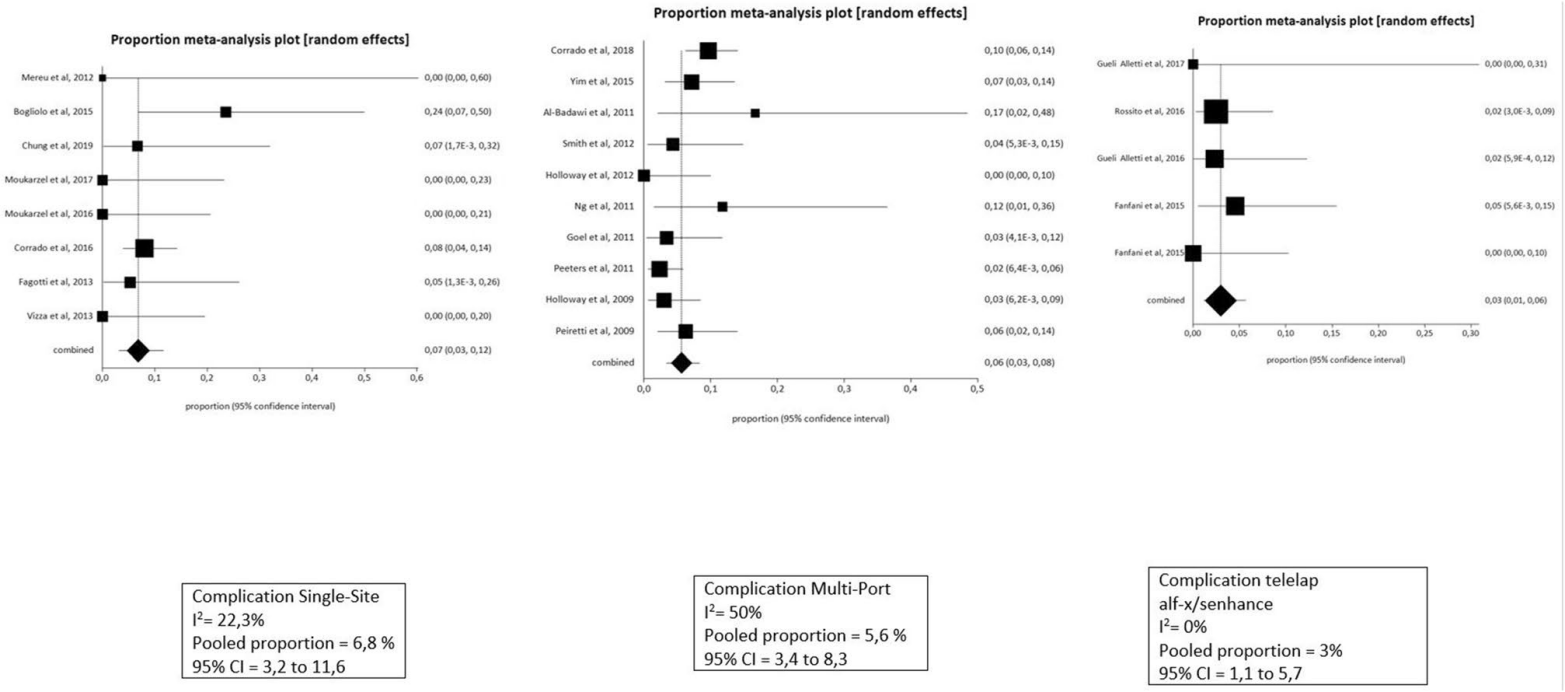
Pooled analysis for complications

Furthermore, as hypothesized, the UMIS technique demonstrated a significant increase in pain management with fewer analgesics needed in various types of surgical procedures when compared to their laparotomic equivalent [70–72] (Figs. 2 and 3).

Indeed, the progressive reduction in the skin incision size is immediately mirrored in the decrease of parietal neuro-muscular injury with concomitantly reduced incisional pain..

As reported by Cianci et al. [47], referred pain was better in the percutaneous approach than in the minilaparoscopic approach (VAS score 3 vs 5 at 24 h after surgery, respectively).

Overlapping results were also shown by Perrone et al. [73] in a multicentric cohort study comparing percutaneous with “classical laparoscopic surgery”.

Finally, since no clinical trials on this topic are currently available, we can conclude that both the percutaneous and minilaparoscopic approaches represent an opportunity to improve patient-referred pain compared to the “classical” minimally invasive approaches in selected cases and experienced hands (Tables 4, 5, 6, and 7).

**Table 4:** Type of complications

	Single Port Group 227 (*n*;%)	Multi Port Group 881 (*n*;%)	Telelap Alf-x/Senhance Group 212 (*n*;%)	Total 1320 (*n*;%)	*p* value
Vascular	3; 1.3%	8; 0.9%	3; 1.4%	14; 1.1%	0.42
Vaginal	1; 0.4%	4; 0.5%	0; –	5; 0.4%	0.55
Urinary	2; 0.9%	2; 0.2%	2; 0.9%	6; 0.5%	0.6
Infectious	5; 2.2%	10; 1.1%	0; –	15; 1.1%	0.19
Thrombotic	2; 0.9%	1; 0.1%	0; –	3; 0.2%	0.41
Neurological	2; 0.9%	0; –	0; –	2; 0.2%	0.14
Bowel	1; 0.4%	2; 0.2%	0; –	3; 0.2%	0.57
Chyle ascites	0; –	1; 0.1%	0; –	1; 0.1%	0.52
Anesthesiological	0; –	2; 0.2%	0; –	2; 0.2%	0.25
Not Specified	0; –	22; 2.5%	0; –	22; 1.7%	0.52
Total	16; 7.0%	52; 5.9%	5; 2.4%	73; 5.5%	0.058

Vascular complication: hemoperitoneum, intra- or post-operative bleeding. Vaginal Complication: vaginal cuff hematoma or dehiscence. Urinary complication: urethral fistula, bladder lesion or bladder fistula. Infectious complications: fever, pelvic abscess, wound infection. Thrombotic complications: pulmonary thromboembolism, deep vein thrombosis. Neurological complications: sciatic pain, lower limb neuropathy. Bowel complications: paralytic ileus, incisional hernia. Anesthesiological complications: respiratory failure, supraventricular tachycardia

**Table 5:** Laparotomic conversions

	Single Port Group 227 (*n*;%)	Multi Port Group 881 (*n*;%)	Telelap Alf-x/Senhance Group 212 (*n*;%)	Total 1320 (*n*;%)	*p* value
Surgical difficulty	1; 0.4%	7; 0.8%	3; 1.4%	11; 0.8%	0.22
Anesthesiological	1; 0.4%	3; 0.3%	4; 1.9%	8; 0.6%	0.02
Intra-operative bleeding	0; –	6; 0.7%	3; 1.4%	9; 0.7%	0.09
Large uterine size	0; –	10; 1.1%	7; 3.3%	17; 1.3%	0.02
Not specified	1; 0.4%	0; –	0; –	1; 0.1%	0.39
Total	3; 1.3%	26; 3.0%	17; 8.0%	46; 3.5%	0.051

Surgical difficulty: poor exposure, aortic nodal staging, bladder lesion, severe adhesion. Anesthesiological complications: hypercapnia

**Table 6:** Surgical outcomes

Variables	Single-port group	Multi-port group	Telelap Alf-x/Senhance Group	*p* value
Operative time (min)	163	181	160	0.528
Estimated blood loss (mL)	62.5	118	50	0.026
Conversion (*n*)	3	26	17	0.051
Complication (*n*)	16	53	5	0.058
Hospital stay (day)	2	1.4	2	1.000
FIGO stage > II (*n*)	2	148	0	0.023

All variables are expressed in median

*Min* minutes, *mL* milliliters, *n* number

**Table 7:** Contingency table

Type of surgery	Hysterectomy	Hysterectomy plus sentinel lymph node	Hysterectomy plus lymphadenectomy	Total
Single-port	5	2	1	8
Multi-port	0	0	10	10
Telelap Alf-x/Senhance	1	0	4	5
Total	6	2	15	23

### Surgical outcomes

In our series, all the papers analyzed showed a comparable median OT, EBL, complication rate, and type of procedures between MIS and UMIS.

Furthermore, even in the setting of advanced surgical procedures, such as pelvic lymphadenectomy, median OT and complications were superimposable to that reported for the standard laparoscopic approach [74–77].

Besides, only “minor complications” (Clavien-Dindo grade 1–2) were reported in our series.

However, all the analyzed reports were referred to high-volume third-level centers for gynecological malignancies, making more difficult the generalization of the obtained results.

Another technical aspect that contributes to the excellent surgical outcomes is the maintenance of the standard laparoscopic triangulation even in the UMIS approach.

Usually, two needlescopic instruments in the left and right flank (2.9 mm of Percuvance ™ or 2.4 mm of Mini-Grip™) and one 5 mm operative suprapubic trocar are positioned in percutaneous approach while three 3 mm trocar are placed, in the same positions, during minilaparoscopic approach [78].

In this scenario, percutaneous and minilaparoscopic surgery may be more feasible and manageable than other single port MIS in which triangulation is lacking [79].

## Weaknesses

### Manipulating tissue and coagulation

According to several authors, the fundamental limitation of percutaneous instrumentation is the limiting of tissue mobilization due to the shaft’s diameter [43]. As a result, percutaneous tools may buckle when treating heavy structures such as massive ovarian masses. In addition, the inefficient lever effect is amplified by the abdominal wall’s high resistance, which amplifies the instrument’s flexion.Even the small size of the instrument’s jaw could negatively impact the correct mobilization of enlarged uteri (> 250 g) or adnexal masses [80, 81] while determining an increased risk of tissue laceration [82].

Finally, as pointed out by several authors, the lack of energy in percutaneous instruments makes multifunction devices recommended, even in cases with relatively low technical difficulty [13, 43].

Consequently, if, on the one hand, an excellent surgical performance with reduced operating times was guaranteed through the use of an integrated energy device, on the other, costs were increased.

### Feeling in managing tissues

Gueli Alletti et al. [42] has highlighted the lack of tissue manipulation feeling as the primary constraint of percutaneous endoscopic instrumentation in a research including 382 patients who received “complex gynecological procedures.”.

Needleoscopic tools are inserted directly into the abdominal cavity losing the smooth glide of the instrument inside the trocar. In this way, the laparoscopic instrument rubbed with all components of the anterior abdominal wall (skin, subcutaneous fat, fascia, muscles, and peritoneum).

This pitfall together with the small and sharp operating tip makes tissue manipulation less sensitive by increasing the risk of tissue tearing if excessive traction is applied [48].

This limitation was particularly evident in the manipulation of soft tissues, such as in lymph node grasping during nodal dissection in endometrial cancer cases [42].

### Review strengths and limitations

There were several limits to our review. First of all, we only considered studies performed at third-level oncological centers. It should be noted that all of the studies included were retrospective in design, and no control groups were included. At the least, the number of described case series is limited. The primary strength of our review was the only complex gynecological surgeries inclusion, hence minimizing the selection bias.

## Conclusions

Even for complicated gynecological procedures, the UMIS techniques proved viable and safe.

Operation durations and problems were significantly decreased compared to “classical” minimally invasive procedures mentioned in the literature.

## Data Availability

On request.
